# Expression of IL-23/Th17-related cytokines in basal cell carcinoma and in the response to medical treatments

**DOI:** 10.1371/journal.pone.0183415

**Published:** 2017-08-22

**Authors:** Cristina Pellegrini, Augusto Orlandi, Gaetana Costanza, Alessandro Di Stefani, Antonella Piccioni, Antonella Di Cesare, Andrea Chiricozzi, Amedeo Ferlosio, Ketty Peris, Maria Concetta Fargnoli

**Affiliations:** 1 Biotechnological and Applied Clinical Sciences, University of L’Aquila, L’Aquila, Italy; 2 Department of Biomedicine and Prevention, Anatomic Pathology, University of Rome Tor Vergata, Rome, Italy; 3 Institute of Dermatology, Catholic University, Rome, Italy; 4 Division of Clinical, Preventive and Oncologic Dermatology, Department of Surgery and Translational Medicine, University of Florence, Florence, Italy; 5 Dermatology Department, University of Pisa, Pisa, Italy; Mie University Graduate School of Medicine, JAPAN

## Abstract

Several immune-related markers have been implicated in basal cell carcinoma (BCC) pathogenesis. The BCC inflammatory infiltrate is dominated by Th2 cytokines, suggesting a specific state of immunosuppression. In contrast, regressing BCC are characterized by a Th1 immune response with IFN-γ promoting a tumor suppressive activity. IL-23/Th17-related cytokines, as interleukin (IL)-17, IL-23 and IL-22, play a significant role in cutaneous inflammatory diseases, but their involvement in skin carcinogenesis is controversial and is poorly investigated in BCC. In this study we investigated the expression of IFN-γ, IL-17, IL-23 and IL-22 cytokines in BCC at the protein and mRNA level and their modulation during imiquimod (IMQ) treatment or photodynamic therapy (PDT). IFN-γ, IL-17, IL-23 and IL-22 levels were evaluated by immunohistochemistry and quantitative Real Time PCR in 41 histopathologically-proven BCCs (28 superficial and 13 nodular) from 39 patients. All BCC samples were analyzed at baseline and 19 of 41 also during medical treatment (9 with IMQ 5% cream and 10 with MAL-PDT). Association between cytokines expression and clinico-pathological variables was evaluated. Higher levels of IFN-γ, IL-17, IL-23 and IL-22 were found in BCCs, mainly in the peritumoral infiltrate, compared to normal skin, with the expression being correlated to the severity of the inflammatory infiltrate. IFN-γ production was higher in superficial BCCs compared to nodular BCCs, while IL-17 was increased in nodular BCCs. A significant correlation was found between IFN-γ and IL-17 expression with both cytokines expressed by CD4+ and CD8+ T-cells. An increase of all cytokines occurred during the inflammatory phase induced by IMQ and at the early time point of PDT treatment, with significant evidence for IFN-γ, IL-23, and IL-22. Our results confirm the role of IFN-γ and support the involvement of IL-23/Th17-related cytokines in BCC pathogenesis and in the inflammatory response during IMQ and MAL-PDT treatments.

## Introduction

Basal cell carcinoma (BCC) is the most common cutaneous malignant neoplasia in light-skinned populations [1, 2] representing a growing public health care problem [3, 2]. BCC is usually characterized by slow growth and low metastatic potential although it might be associated with severe morbidity due to its ability to enlarge progressively and to destroy adjacent tissues [4].

There is good evidence that BCC is an immunogenic tumor [5] The intra- and peritumoral inflammatory infiltrate of the BCC microenvironment is characterized by a strong predominance of T lymphocytes, mainly CD4+ T-helper (Th) cells and CD4+/CD25+/FoxP3+ T regulatory (regs) cells, with a variable number of CD8+ T cytotoxic/suppressor (Tc) cells, Langerhans cells, natural killer (NK) cells and immature dendritic cells (DC) [6, 7, 8, 9, 10]. Few studies investigated the role of infiltrating T lymphocytes and pro-inflammatory cytokines secreted in the BCC microenvironment, and it is still unclear whether the inflammatory reaction promotes tumor growth or exerts anti-tumor activity [6, 8, 10]. Both an increased expression of interferon-(IFN) associated genes inducing a Th1 response and local production of cytokines which favor a Th2 environment have been described in BCC [5, 6, 8, 9, 10]. Interestingly, high levels of IFN-γ were observed in regressing BCC, indicating an enhanced antitumor Th1 immune response [6, 11].

The IL-23/Th17-related cytokines (IL-17, IL-23, IL-22) have been shown to play a significant role in cutaneous immune-mediated inflammatory diseases, including psoriasis, allergic contact dermatitis and atopic dermatitis [12, 13, 14], but their involvement in skin carcinogenesis is controversial and poorly investigated in BCC [7, 10, 14, 15]. IL-17, mainly produced by Th17 cells, exerts its pro-inflammatory function by inducing neutrophil recruitment, expansion and function [14]. Moreover, it enhances DC maturation, T cell priming, and cellular (e.g., fibroblasts, macrophages, epithelial cells) production of inflammatory mediators [16]. Two recent studies suggested a role for IL-17 in BCC pathogenesis showing a high number of infiltrating IL-17+ lymphocytes in the BCC peritumoral area and IL-17-induced proliferation and migration of human BCC cell lines [10, 14]. IL-23 is a pro-inflammatory cytokine, mainly produced by activated DC and macrophages [17], that actively participates in cellular immunity driving the expansion, stabilization and survival of Th17 cells [18]. The influence of IL-23 as protumoral or antitumoral cytokine in cancer is still undefined, being reported both to promote tumor growth [17] and to induce apoptosis [19] and it is relatively unexplored in BCC tumors. IL-22 is a member of the IL-10 family of cytokines and represents an important effector of activated Th22, Th1, Th17 cells, as well as CD8+ T cells (Tc17 and Tc22), NK cells and myeloid cells. It acts on epithelial cells mediating a cellular inflammatory response and exhibiting antiapoptotic and tumorigenic functions [20]. A tumor proliferating role has been recently shown to be induced by IL-22 in BCC cell lines [10].

Although surgical excision is the standard of care for BCC, medical treatments as imiquimod (IMQ) and topical photodynamic therapy (PDT) are recommended for low-risk superficial and small nodular BCC [21, 22]. IMQ is a potent agonist of Toll-like receptors (TLR) 7 and 8 expressed by both innate and adaptive immune cells [22, 23]. TLR7/8 activation promotes anti-cancer host defense through the secretion of pro-inflammatory cytokines and chemokines, including IFN-α, IFN-γ and tumor necrosis factor (TNF)-α, acting on DC, macrophages and T cells, especially of the Th1 subset [24]. This inflammatory milieu activates cytotoxic functions of CD8+ T cells and promotes selective apoptosis of tumor cells [25]. Recent evidences suggest the involvement of the IL-23/Th17 pathway in the mechanism of action of IMQ [26, 27].

PDT exerts its action through light activation of a photosensitizer (5’-aminolevulinic acid or methyl aminolaevulinate, MAL) in the presence of oxygen leading to release of reactive oxygen species that cause a selective destruction of the target tissue [28]. Besides a direct anti-tumor effect, PDT has been hypothesized to indirectly induce anti-tumor immunity [29, 30].

In this context, aim of our study was to investigate expression of IFN-γ, IL-17, IL-23 and IL-22 in BCC and their modulation during IMQ 5% cream or MAL-PDT treatment.

## Materials and methods

### Patients’ selection

Patients were recruited at the outpatient clinic of the Department of Dermatology, University of L’Aquila, Italy, from September 2009 to March 2012. All patients older than 18 years of age with histopathologically-proven BCC were eligible for this study. Two clinical BCC subtypes were considered, i.e., superficial BCC (sBCC) and nodular BCC (nBCC). Patients with aggressive BCC subtypes, recurrent BCC or with Gorlin syndrome were excluded.

At the first visit, clinical and dermoscopic photographs of the lesions were taken and tumor size was measured. Presence or absence of pigmentation was recorded. Normal skin of patients attending the Plastic Surgery Department of our Hospital during the same time period was used as control.

The study was approved by the Local Ethics Committee (ASL L’Aquila-Teramo). Written informed consent was obtained from all participants, and the study was conducted according to the Declaration of Helsinki Principles.

### Treatment and skin sampling

BCC lesions were either surgically excised or, if clinically indicated, treated with IMQ 5% cream or topical MAL-PDT. For surgically excised BCCs, a 3-mm intra-tumoral punch biopsy specimen was obtained and stored in RNA later solution at -20°C for molecular analysis. The remaining tissue was formalin-fixed and paraffin-embedded for conventional histopathology. For BCCs treated with medical therapy, one 4-mm intra-tumoral punch biopsy specimen and, if possible, an additional 3-mm punch biopsy specimen, were collected at baseline for histopathological evaluation and mRNA analysis, respectively.

Treatment with IMQ 5% cream or topical MAL-PDT was administered according to approved protocol. IMQ 5% cream was applied 5 times weekly, in the evening, on the BCC lesion until the occurrence of the inflammatory reaction or for a maximum of 12 weeks. A cycle of MAL-PDT with two sessions at 1-week interval was performed. MAL (Metvix cream^®^, Galderma, Paris, France) was applied over the entire BCC under occlusion for three hours after removal of crusts and scales for sBCC or gentle debulking for nBCC and the lesion was then illuminated with red (635 ± 18 nm) light at a dose of 37J/cm^2^ from a diode lamp (Aktilite^®^CL128; Photocure ASA, Oslo, Norway) for 7 minutes and 40 seconds at the distance of 8 cm. Response rate for both treatments was evaluated clinically and dermoscopically at 3 months after treatment and classified as complete response (CR), partial response (PR) and lack of response (NR). In order to investigate therapy-based modulation of cytokines, additional tumor biopsy specimens were collected at scheduled time points during treatment. For BCCs treated with IMQ the samples were collected during the inflammatory reaction; for lesions treated with MAL-PDT, an early timepoint (30 minutes to 2 hours after the first session) and a late timepoint (1 week to 3 months after the first session) were chosen according to a previous study [31], demonstrating that the inflammatory response was immediate and intense after PDT irradiation, while no altered features were evident 2, 4, or 8 weeks after PDT.

### Histopathological and immunohistochemical study

For histopathological evaluation, 4-μm-thick tissue sections were routinely stained with hematoxylin and eosin. All cases were independently reviewed by two certified pathologists (A.O., A.D.S.) for assessment of the following histopathological criteria: histopathological variant according to standardized World Health Organization [32], and presence or absence of severe solar dermal elastosis [33]. Moreover, peritumoral inflammatory infiltrate was categorized according to guidelines by Kossard et al. [34] as mild, moderate or severe, also counting the number of cells per mm^2^. After deparaffinization and blocking of endogenous peroxidase activity with 0.2% H_2_O_2_ (20 min), immunostaining with goat polyclonal anti-human IL-17 (1:100; R&D Systems, Minneapolis, MN), rabbit polyclonal anti-IL-23 (1:200; Lifespan Biosciences, Seattle, WA), anti-IFN-γ (1:400; Abcam, Cambridge, UK) and anti-IL-22 (1:200; Abcam) were incubated with BCC slides, followed by universal Dako system (Universal LSAB^™^+ Kit/HRP, Rabbit/Mouse/Goat) for IL-17 antibody, and secondary goat-anti rabbit for the other antibodies. Amino ethyl carbazol was used as final chromogen. All immunohistochemical procedures were performed using positive and negative internal controls as reported [35]. For double staining of IL-17 or IFN-γ with CD4 or CD8, the slides were incubated with monoclonal mouse anti-human CD4 (7 μg/mL; Dako) and monoclonal mouse anti-human CD8 (0.51 μg/mL; Dako) using EnVision^™^DuoFLEX Doublestain System (Dako, code K6807). Diaminobenzidine was used for anti-CD4 and anti-CD8 detection and Liquid Permanent Red for IL-17 and IFN-γ.

Semiquantitative immunoreaction for IL-17, IL-23 and IFN-γ [36] and for IL-22 [37] was estimated by two of the authors (A.O., G.C.), as previously reported.

### RNA isolation and quantitative Real Time PCR

Total RNA was extracted from 3-mm skin biopsy specimens using the RNeasy Mini Kit (Qiagen, Chatsworth, CA), according to manufacturers’ protocols. Biopsies were processed using a rotor-stator tissue homogenizer (Precellys 24, Bertin Technologies) for two cycles of 30 s at 5900 rpm. Quality and quantity of extracted RNA was measured by calculation of the optical density with an ND-1000 Spectrophotometer (NanoDrop, Wilmington, DE). In order to detect mRNA expression levels of cytokines, quantitative real-time PCR was performed. Briefly, using mRNA as template, single-stranded cDNAs were generated from 1 μg of total RNA by the High Capacity cDNA Reverse Transcription kit (Applied Biosystem-Life technologies, Milan, Italy) according to manufacturer’s directions. IFN-γ, IL-23, IL-17 and IL-22 mRNA levels were measured by real-time quantitative PCR on the 7500 Fast real-time PCR system (Applied Biosystems-Life technologies) using Taqman^®^ Technology with the following assays, containing validated PCR primers and TaqMan MGB probes (6FAM-labeled): IFN-γ Hs00174143_m1; TNF-α Hs00174128_ml; IL-23A Hs00372324_ml; IL-17Hs00174383_m1 (this assay amplifies the IL17A isoform); IL-22 Hs00220924_m1 (Applied Biosystems-Life technologies). The thermal cycling conditions were as follows: 2 min at 95°C, followed by 40 cycles of 95°C for 15 s and 59°C for 45 s. Cytokine expression levels were reported as relative units with respect to mRNA levels of *GAPDH* gene used as reference gene to normalize each sample. Each gene was analyzed in triplicate. Relative quantitative evaluation of mRNA was performed by the comparative ΔΔCt method.

### Statistical analysis

Univariate analysis of relationship between demographic, clinical and histopathological features was performed by Spearman’s rank correlation test. Immunohistochemical semiquantitative data were analyzed by means of Student’s *t*-test or ANOVA. Results of molecular analysis are given as medians; mann-Whitney test, paired t test, non parametric 2-tailed Wilcoxon test were used, as appropriate. Associations between IFN-γ, IL-17, IL-22, IL-23, and clinico-pathological variables were analyzed using Pearson’s test (χ^2^ test) or Fisher extract test. In general, p values less than 0.05 were considered statistically significant. All statistical analysis was performed using the statistical package SPSS13.0 (SPSS Incorporated, Chicago).

## Results

### Clinico-pathological characteristics of BCC and control samples

Forty-one BCC samples were collected from 39 patients (24 M, 15 F; median age at diagnosis: 68 years, range: 37–87 years). The anatomic location was the trunk in 29/41 (70.7%) BCCs, the head/neck region in 11/41 (26.8%) and the extremities in 1/41 (2.4%). Pigmentation was present in 16/41 (39%) BCCs. After histopathological examination, 28/41 (68.3%) lesions were categorized as sBCC and 13 (31.7%) as nBCC. Seventeen of 41 (41.5%) BCCs showed solar dermal elastosis, whereas 24 (58.5%) did not. The peritumoral inflammatory infiltrate at baseline was mild in 6 of 41 (14.6%) samples, moderate in 16 of 41 (39.0%) and severe in 19 of 41 (46.4%). Clinico-pathological features of BCCs are listed in Table 1.

**Table 1:** Clinico-pathological features and type of treatment for BCC lesions included in the study.

No. BCC	*Anatomic location*	*Pigmentation*	*Histological Subtype*	*Peritumoral inflammatory infiltrate*	*Solar Elastosis*	IHC analysis	Molecular analysis	Treatment	Response
**1**	Trunk	No	Nodular	Moderate	No	•		PDT	NR
**2**	Head/Neck	No	Nodular	Moderate	Yes	•	•	PDT	CR
**3**	Trunk	No	Nodular	Moderate	No	•		IMQ	RC
**4**	Trunk	No	Superficial	Moderate	No	•		PDT	NR
**5**	Trunk	No	Nodular	Severe	No	•	•	PDT	CR
**6**	Trunk	No	Nodular	Severe	No	•	•	PDT	CR
**7**	Trunk	No	Nodular	Moderate	No	•		IMQ	CR
**8**	Trunk	No	Superficial	Mild	No	•		IMQ	PR
**9**	Head/Neck	No	Nodular	Moderate	Yes	•		IMQ	CR
**10**	Trunk	Yes	Nodular	Severe	No	•		Surgery	na
**11**	Trunk	Yes	Superficial	Severe	No	•	•	IMQ	CR
**12**	Trunk	No	Nodular	Moderate	No	•	•	IMQ	CR
**13**	Trunk	No	Nodular	Mild	No	•		PDT	CR
**14**	Trunk	No	Superficial	Severe	Yes	•	•	IMQ	PR
**15**	Head/Neck	No	Nodular	Severe	No	•	•	Surgery	na
**16**	Trunk	No	Superficial	Mild	No	•	•	IMQ	CR
**17**	Trunk	No	Superficial	Severe	Yes	•	•	PDT	CR
**18**	Trunk	Yes	Superficial	Severe	Yes	•	•	IMQ	CR
**19**	Trunk	No	Nodular	Moderate	Yes	•	•	PDT	PR
**20**	Trunk	No	Superficial	Moderate	No	•	•	PDT	CR
**21**	Extremities	Yes	Nodular	Moderate	Yes	•	•	Surgery	na
**22**	Trunk	No	Superficial	Moderate	No	•	•	PDT	NR
**23**	Trunk	Yes	Nodular	Severe	No	•		Surgery	na
**24**	Head/Neck	No	Nodular	Severe	Yes	•		Surgery	na
**25**	Trunk	Yes	Superficial	Moderate	No	•		Surgery	na
**26**	Head/Neck	Yes	Nodular	Severe	Yes	•		Surgery	na
**27**	Trunk	Yes	Superficial	Moderate	No	•		Surgery	na
**28**	Trunk	Yes	Nodular	Severe	Yes	•		Surgery	na
**29**	Head/Neck	Yes	Superficial	Severe	No	•		Surgery	na
**30**	Head/Neck	No	Nodular	Severe	Yes	•		Surgery	na
**31**	Head/Neck	No	Nodular	Mild	Yes	•	•	Surgery	na
**32**	Head/Neck	No	Nodular	Mild	Yes	•	•	Surgery	na
**33**	Trunk	No	Nodular	Severe	No	•	•	Surgery	na
**34**	Trunk	No	Superficial	Mild	No	•	•	Surgery	na
**35**	Trunk	Yes	Nodular	Moderate	No	•	•	Surgery	na
**36**	Trunk	Yes	Nodular	Severe	No	•	•	Surgery	na
**37**	Head/Neck	Yes	Nodular	Severe	Yes	•	•	Surgery	na
**38**	Head/Neck	Yes	Nodular	Moderate	Yes	•	•	Surgery	na
**39**	Trunk	Yes	Nodular	Moderate	Yes	•	•	Surgery	na
**40**	Trunk	Yes	Nodular	Moderate	Yes	•	•	Surgery	na
**41**	Trunk	Yes	Nodular	Severe	No	•	•	Surgery	na

BCC, basal cell carcinoma; IHC immunohistochenistry; PDT photodynamic therapy; IMQ, imiquimod; CR complete response; PR partial response; NR non responder; na not available. BCCs evaluated by immunohistochemistry and quantitative Real Time PCR are indicated in the corresponding column with a black dot (•)

As control, normal skin from 8 patients (3 M, 5 F; median age: 40 years, range: 27–60) was analyzed. In detail, 5 of 8 (62.5%) skin specimens were excised from the trunk, 2 of 8 (25%) from the head/neck region and 1 of 8 (12.5%) from the extremities.

The Spearman analysis showed a positive correlation between localization of BCCs on head and neck areas and the presence of solar elastosis (r_s_ = 0.576, p<0.01). A positive correlation was also observed between pigmentation and peritumoral inflammation: pigmented BCCs were significantly associated to a moderate/severe inflammatory infiltrate (r_s_ = 0.357, p<0.01).

### Protein expression levels of IL-17, IL-23, IFN-γ and IL-22 in BCC samples

**Fig 1:**
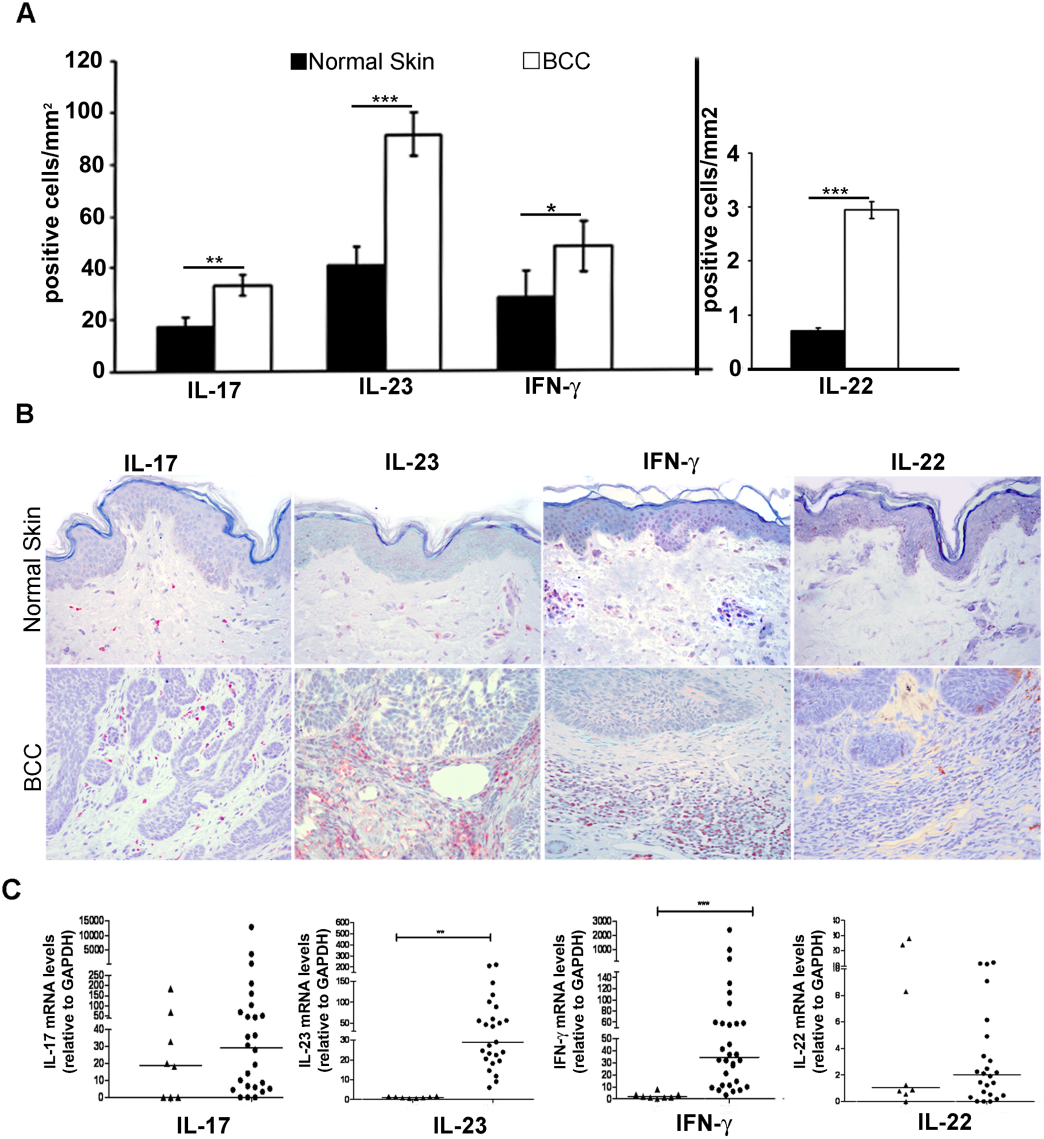
Immunohistochemical and mRNA expression of IL-17, IL-23, IFN-γ and IL-22 in normal skin and BCC. (A) Bar graphs showing cytokines+ inflammatory cells per mm^2^ in normal skin compared to BCC. *p<0.05; **p<0.01; ***p<0.001; (B) Immunodetection of all cytokines was significantly higher in BCC samples compared to normal skin, and mainly expressed in inflammatory cells of peritumoral infiltrate; (C) Significantly elevated IL-23 and IFN-γ mRNA expression in BCCs as compared to normal skin. Increased expression of IL-17 and IL-22 mRNA in BCCs, as compared to control samples.**p<0.01; ***p<0.001.

Immunohistochemical evaluation was carried out in all 41 BCC samples and 8 control samples. Expression levels of all 4 tested cytokines were significantly higher in BCC samples compared to normal skin, and they were mainly expressed in the peritumoral inflammatory cells (Fig 1A and 1B). IL-17 was negative in BCC cells and control skin, but expressed in the cytoplasm of inflammatory cells with a granular staining (p<0.01). IL-23 was strongly expressed in dermal inflammatory cells, but focally weakly positive in BCC cells and in keratinocytes of normal skin (p<0.01). IFN-γ was not or only scantly expressed in BCC cells, while it was abundantly expressed in the cytoplasm of peritumoral inflammatory cells (p<0.05). IL-22 immunoreaction was negative in BCC but positive in inflammatory cells of the dermal infiltrate (p<0.01).

**Fig 2:**
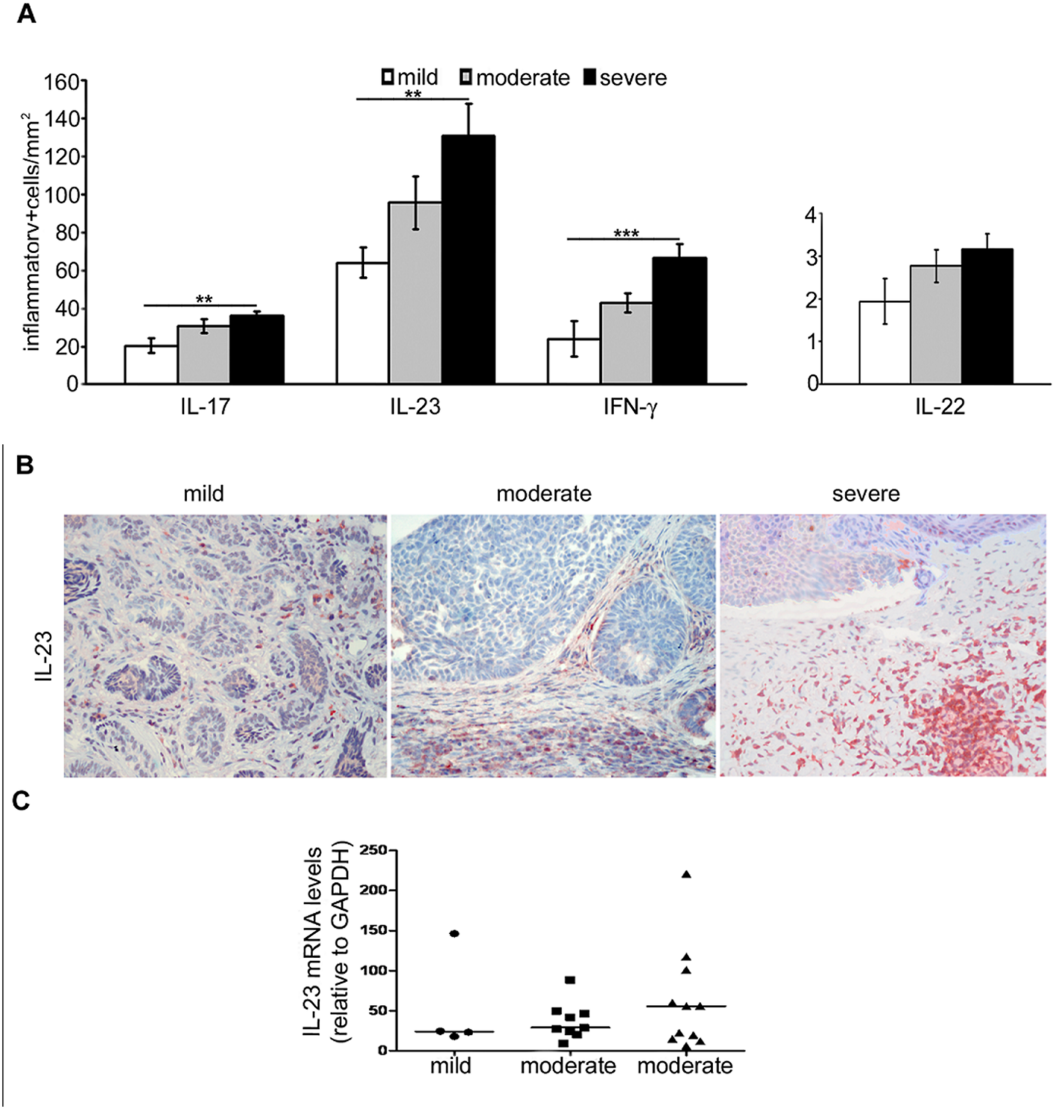
Immunohistochemical and mRNA expression of IL-17, IL-23, IFN-γ and IL-22 varies according to severity of the inflammatory infiltrate in BCC. (A) Graph showing cytokine levels in BCC, considering the severity of the inflammatory infiltrate. Statistically significant higher levels of IL-17, IL-23 and IFN-γ expression were observed with the increasing amount of peritumoral inflammatory infiltrate and the trend was similar for IL-22 expression. **p<0.01; ***p<0.001; (B) Exemplificative images of increasing IL-23 immunostaining in BCC samples associated to absent/mild, moderate and severe inflammation; (C) Variation of IL-23 mRNA expression levels according to the amount of the peritumoral inflammatory infiltrate.

**Fig 3:**
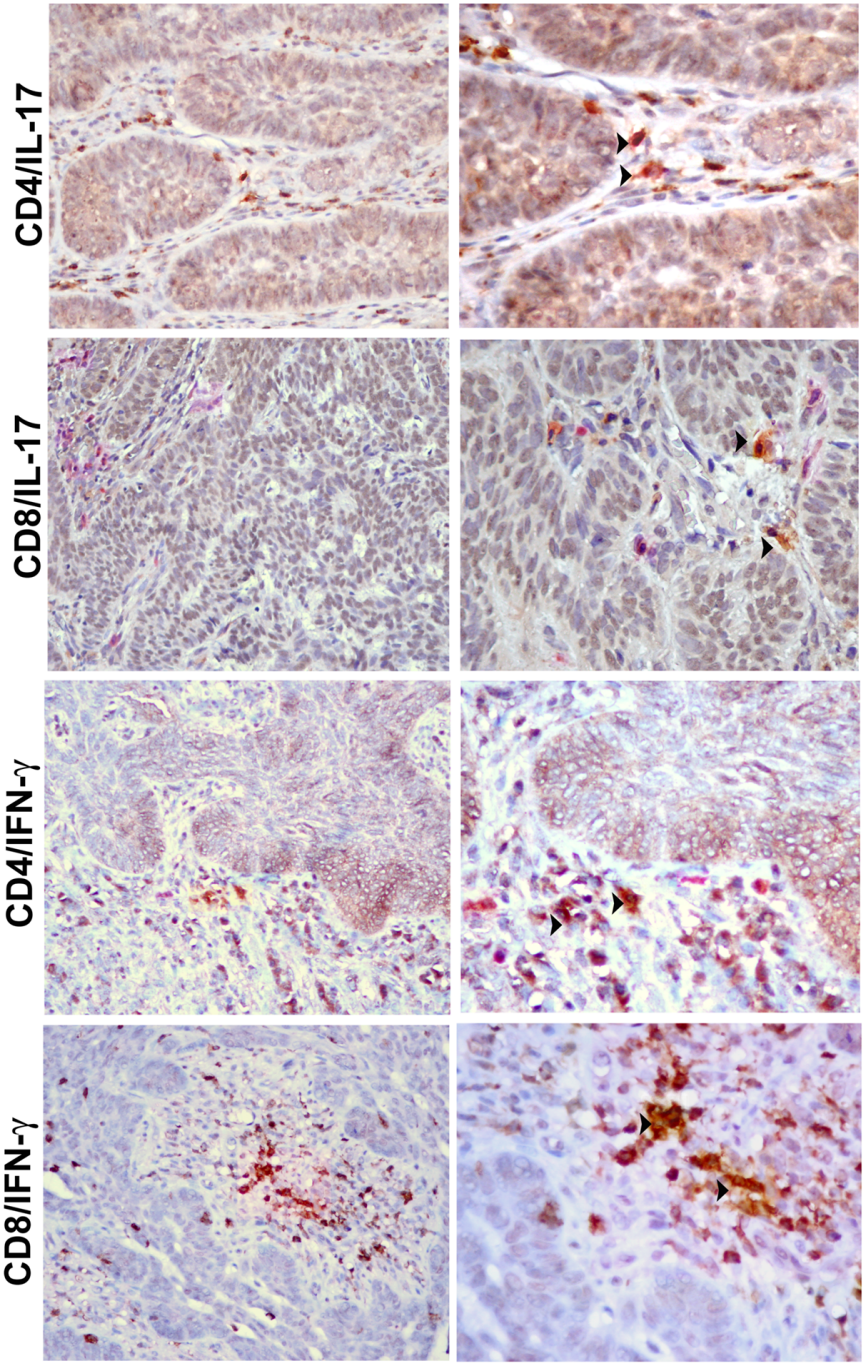
Double staining of IL-17 or IFN-γ with CD4 or CD8. (A) Double-staining of IL-17 (red) and CD4+ (brown) inflammatory cells and (B) of IL-17+ (red) and CD8+ (brown) cells. (C) Double-staining of IFN-γ (red) and CD4+ (brown) inflammatory cells and (D) of IFN-γ (red) and CD8+ (brown) inflammatory cells. Arrowheads indicate double-positive cells. Left panel magnification: 200×; right panel magnification: 400×.

Levels of IL-17, IL-23 and IFN-γ increased with the severity of the peritumoral inflammatory infiltrate (p<0.01 for IL-17, IL-23 and IFN-γ) and the trend was similar for IL-22 expression (Fig 2A). The Spearman analysis confirmed a positive correlation between expression of IL-17 or IL-23 and increase of the peritumoral inflammatory infiltrate (p<0.05) and showed that high levels of IL-17 expression correlated with high levels of IFN-γ expression (p<0.01). Double-staining demonstrated that IL-17 was expressed by CD4+ as well as by CD8+ inflammatory cells. Likewise, a fraction of CD4+ or CD8+ cells co-expressed IFN-γ (Fig 3).

With regard to the histopathological subtype, IFN-γ expression was significantly higher in sBCC as compared to nBCC (p<0.03) (S1 Fig). A similar increase was observed for IL-23 although not significant. Conversely, there was a trend for a higher expression of IL-17 and IL-22 in nBCC as compared to sBCC.

No correlation was found between immunoexpression of cytokines and anatomical site of BCC (r_s_ = 0.116 for IFN-γ, r_s_ = 0.001 for IL-17, r_s_ = 0.369 for IL-23, r_s_ = -0.055 for IL-22; all having p>0.05) or absence/presence of solar elastosis (r_s_ = 0.114 for IFN-γ; r_s_ = -0.155 for IL-17; r_s_ = -0.0948 for IL-23, r_s_ = 0.139 for IL-22; all having p>0.05).

### mRNA expression analysis of IL-17, IL-23, IFN-γ and IL-22 in BCC

At baseline, mRNA expression of IL-17, IL-23, IL-22 and IFN-γ was determined in 25 of 41 (60.9%) BCC tumor specimens and in all 8 control samples. Overall, we observed that IL-23 and IFN-γ mRNA expression was elevated in all BCC samples as compared to normal skin (p<0.01 for IL-23 and IFN-γ); in detail, we observed a 26-fold increase of IL-23 and a 19-fold increase of IFN-γ in BCCs (Fig 1C). The expression of IL-17 and IL-22 mRNA was slightly elevated in BCCs as compared to control samples.

Analysis of cytokine expression levels according to the peritumoral inflammatory infiltrate showed that mRNA levels of all Th17-related cytokines (IL-17, IL-23 and IL-22) increased with the amount of the inflammatory infiltrate. As an example, IL-23 profile is illustrated in Fig 2C. The median expression profile of IFN-γ mRNA was comparable among the three subgroups of the inflammatory infiltrate (Kruskal-Wallis coefficient to compare medians is = 0.54 with p = 0.74).

With regard to BCC subtypes, quantitative RT-PCR analysis revealed that nBCCs expressed higher IL-17 mRNA levels as compared to sBCC (p = 0.02), with a 7-fold increase. A trend for increased mRNA levels of IL-23 and IL-22 was shown in nBCCs vs sBCCs. Finally, IFN-γ transcript levels were comparable between the two BCC subtypes.

No difference in mRNA levels of all studied cytokines was found when BCCs with solar elastosis were compared to BCCs without solar elastosis and for different sites (head/neck vs trunk/extremities).

### Immunoexpression and mRNA levels of IL-17, IL-23, IL-22 and IFN-γ before and during treatment of BCC with IMQ 5% cream or MAL-PDT

A total of 19 BCCs were treated with medical treatments, 9 with IMQ 5% cream and 10 with MAL-PDT (Table 1). For BCCs treated with IMQ 5% cream, the overall percentage of CR was 77.7% (7/9) while 22.3% (2/9) showed PR. For lesions treated with MAL-PDT, 70% (7/10) achieved CR, 10% (1/10) PR and 20% (2/10) showed lack of response.

For histopathological and immunohistochemical investigations, we evaluated all 19 BCC samples treated with IMQ 5% or MAL-PDT; in this last case, 7 biopsies were taken at an early time point, and 4 at a late time point.

**Fig 4:**
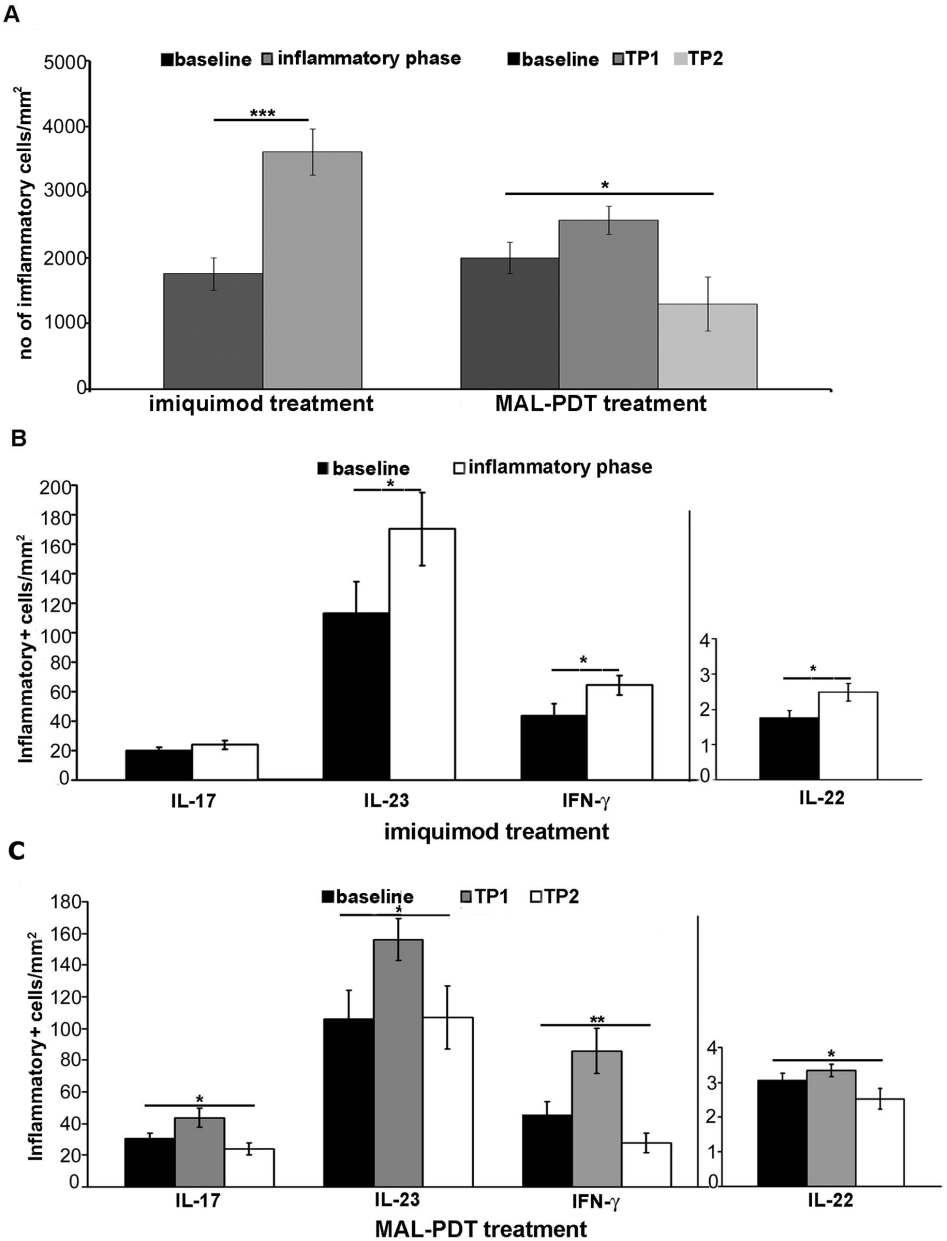
Inflammatory cells and expression of IL-17, IL-23, IL-22 and IFN-γ before and during treatment with IMQ 5% cream or MAL-PDT. (A) Number of inflammatory cells increased significantly during the inflammatory phase of IMQ treatment (p<0.01) and the early time point of MAL-PDT treatment, while decreased at the late time point (*p<0.05). (B) Cytokine levels in BCC before treatment and during the inflammatory phase of IMQ 5% treatment. ANOVA, *p<0.05; **p<0.01 (C) Cytokine levels in BCC at baseline, at the early and late timepoints of MAL-PDT. *p<0.05. *TP1*, early time point; *TP2*, late time point.

The number of peritumoral inflammatory cells significantly increased during the inflammatory phase of IMQ treatment (p< 0.01) and at the early time point of MAL-PDT (p<0.05), while decreased at the late PDT time point (Fig 4A). BCCs treated with IMQ 5% showed a significant increase of IL-23, IFN-γ and IL-22 levels in peritumoral inflammatory cells (p<0.05), whereas an increasing trend was observed for IL-17 (Fig 4B). In cases treated with MAL-PDT, we observed increasing levels of all 4 cytokines at the early time point compared to baseline, followed by decreasing levels at the late timepoint, with a characteristic bell-shaped distribution (p<0.05 for IL-17, IL-23 and IL-22; p<0.01 for IFN-γ; Fig 4C).

For molecular analysis of cytokine mRNA expression levels, we analyzed 6 of the 19 treated lesions, 3 with IMQ 5% cream and 3 with MAL-PDT. In IMQ-treated BCCs, we observed higher mRNA levels for IL-17 and IL-23 during the inflammatory phase than at baseline while no difference was observed for IL-22 and IFN-γ (S2 Fig). mRNA levels of all tested cytokines were increased at the early timepoint of MAL-PDT as compared to baseline followed by a decrease at the late timepoint (S3 Fig), as observed with immunostaining. For both treatments, statistical analysis was not performed because of the small number of cases.

## Discussion

In this study, we investigated the expression of IFN-γ and IL-23/Th17-related cytokines, IL-17, IL-23 and IL-22, in BCC and their modulation during IMQ 5% cream or MAL-PDT treatment. In our samples, BCC microenvironment was characterized by a moderate to severe inflammatory infiltrate expressing high levels of IFN-γ, IL-17, IL-23 and IL-22 cytokines, both at the protein and mRNA levels, with the expression being correlated to the severity of the inflammatory infiltrate. IFN-γ production was higher in sBCC compared to nBCC, while IL-17 resulted increased in nBCC. We observed a significant correlation between IFN-γ and IL-17 expression and both cytokines were expressed by CD4+ and CD8+ T-cells. Finally, an increase of all cytokines occurred during the inflammatory reaction induced by IMQ and at the early time point after MAL-PDT treatment.

The cytokine profile of BCC tumors has been previously described, mainly focusing on Th1 (IFN-γ, IL-2, TNF-β) and Th2 (IL-4, IL-5, IL-6, IL-10) signals [5, 6, 8, 9, 10]. The inflammatory infiltrate seems to be dominated by Th2 cytokines, suggesting a specific state of immunosuppression [6, 8, 9]. In contrast, regressing BCC tumors are characterized by a Th1-mmune response. A significant increase of IFN-y and IL-2 mRNA was indeed reported following experimentally-induced tumor regression [11]. Similarly, high IFN-γ and IL-2 mRNA expression were shown to strongly correlate with T-cell marker CD3 in BCCs that showed histological regression [6]. In our cases, we observed a significant over-expression of IFN-γ in BCC compared to normal skin, and confirmed that the source of this cytokine is the inflammatory infiltrate surrounding the tumor. In addition, we demonstrated that both CD4+ and CD8+ T cells infiltrating the BCC produce IFN-γ. Our results are in line with Kaporis et al (2007) demonstrating abundant CD8+ T cells and increased expression of IFN-associated genes in the BCC immune microenvironment, suggesting a host antitumor response [7].

The involvement of IL-23/Th17-related cytokines in tumor immunity as pro- or anti-tumor mediators remains undefined [38]. The pro-tumorigenic effects have been experimentally demonstrated in mouse models [17, 39; 40, 41] and in different human cancers, including gastric [42, 43, 44], colon [45], hepatic [46] and pancreatic cancers [47], and myeloid leukemia [48]. Induction of angiogenesis and of tumor-promoting cytokines, as IL-6 and IL-8, might explain the pro-tumoral effect [49]. On the other hand, evidences for the tumor suppressive function of the IL-23/Th17 axis have been described in B-acute lymphoblastic leukemia [19], myelodysplastic syndromes [50], hepatocellular carcinoma [51], in ovarian [52] and prostate [53] cancers. The anti-tumor effects seem to be mediated by multiple pathways, including inhibition of T regs, induction of cytotoxic activity and the synergic action with Th1 response [15].

Concerning skin cancers, high levels of IL-17 and IL-23 have been reported in melanoma [54], while few studies investigated the IL-23/Th17 axis in BCC [7, 8, 10, 14]. A higher number of tumor infiltrating IL-17+ lymphocytes was detected in BCC as compared to squamous cell carcinoma and melanoma 14. Overexpression of IL-12/23 was reported by Kaporis et al. (2007) in the inflammatory infiltrate of nBCC [7]. Nardinocchi et al (2015) described a high number of IL-17+ and IL-22+ cells in the BCC and SCC peritumoral infiltrate; they also demonstrated that IL-17 and IL-22 promote proliferation and migration of BCC and SCC cell lines and induce tumor growth in SCC tumor xenografts [10]. In line with these studies, our results reported elevated IL-17, IL-23 and IL-22 expression in BCC tumors, confirming the involvement of IL-23/Th17 cytokines in the BCC immune microenvironment.

Our results demonstrated that IL-17 was expressed by both CD4+ and CD8+ T cells and correlated to IFN-γ production. The presence of tumor-specific CD8+ T cells producing IL-17 in the BCC infiltrate might suggest a cytotoxic IL-17-mediated immune response. Hinrichs et al. (2009) demonstrated that IL-17 secreting CD8+ T cells are converted into IFN-γ-producing effector T cells and displayed enhanced antitumor immunity in murine B16 melanoma [55]. A tumor-suppressive activity through the synergistic stimulating action of IL-17 and IFN-γ cytokines on cytotoxic CD8+ T cells has been previously reported in a large sample of human ovarian cancer [52]. In addition, both IL-17 and IFN-γ were involved in the IL-23-induced antitumor immunity in mouse tumor models of different cancers [56, 57, 58]. These results support a potential synergistic immune response for IL-17 and IFN-γ through the dynamic evolution and plasticity of Th17 cells.

Cytokine expression profile according to histological BCC subtype was reported in only two studies with inconsistent results [7, 14]. No significant differences in IFN-γ expression were observed between non-aggressive (sBCC and nBCC) and aggressive BCCs (micronodular and infiltrative) [7] and a similar number of IL-17+ T cells was present in different BCC variants [14]. We found higher IFN-γ expression in sBCCs and higher IL-17 in nBCCs. Further studies on larger samples are needed to clarify this association.

Our results demonstrated increased levels of IFN-γ, IL-23, IL-22 and IL-17 during IMQ and PDT treatments supporting that their antitumoral effect might be exerted through the activation of both Th1 and IL-23/Th17 axis. Evidences for Th1 and Th17 involvement following IMQ treatment were previously documented [26, 59] while no previous studies investigated the IL-23/Th17 signals following MAL-PDT. Topically applied IMQ was shown to aggravate a psoriatic plaque and to induce *de novo* psoriasis-like skin lesions in mice through activation of the IL-23/Th17 axis [26]. Yokogawa et al. (2013) reported overexpression of IFN-γ, IL-12, IL-23 and IL-17 at the regressing SCC tumor site following IMQ application in mice [27]. On the other hand, PDT response was associated with a gradual increase of peritumoral CD3+ lymphoid cells, mainly CD4+, and of CD68+ macrophages as well as with an increased expression of proinflammatory cytokines, as IL-2, IL-6, IL-10 and TNF [30, 31, 60, 61, 62].

We investigated the involvement of the IL-23/Th17 pathway in BCCs both at the protein and mRNA levels with consistent results. Our results could have been expanded by deeply analyzing the source of expression of the different Th1 and Th17 cytokines in the BCC inflammatory infiltrate although this was beyond the aim of this study. A larger sample size of BCCs during IMQ and MAL-PDT treatment is needed to better combine protein and mRNA data and to correlate experimental findings with clinical response.

## Conclusions

In conclusion, our results confirm the role of IFN-γ and support the involvement of IL-23/Th17-related cytokines in BCC pathogenesis and in the response to IMQ ad MAL-PDT treatments. Enhancing the understanding of BCC pathogenic mechanism, the management of this disease could be further optimized.

## Supporting information

S1 Fig Cytokine expression according to histopathological subtype. IFN-γ expression was significantly higher in sBCC as compared to nBCC (*p<0.05). A nonsignificant increase was also observed for IL-23. A trend for a higher expression of IL-17 and IL-22 was observed in nBCC compared to sBCC.
(TIF)

S2 Fig mRNA expression levels of IL-17, IL-23, IL-22 and IFN-γ before and during the inflammatory phase of IMQ 5% treatment. mRNA levels for IL-17 and IL-23 were higher during the inflammatory phase than at baseline while no difference was observed for IL-22 and IFN-γ.
(TIF)

S3 Fig mRNA expression levels of IL-17, IL-23, IL-22 and IFN-γ before and during treatment with MAL-PDT. mRNA levels of all tested cytokines were increased at the early timepoint of MAL-PDT as compared to baseline followed by a decrease at the late timepoint. *TP0*, *baseline; TP1*, early time point; *TP2*, late time point.
(TIF)
