# Supplementary figures and images for: Expression of IL-23/Th17-related cytokines in basal cell carcinoma and in the response to medical treatments
Source: PLoS One. 2017 Aug 22;12(8):e0183415. doi: 10.1371/journal.pone.0183415 (PMC5567915; doi:10.1371/journal.pone.0183415)

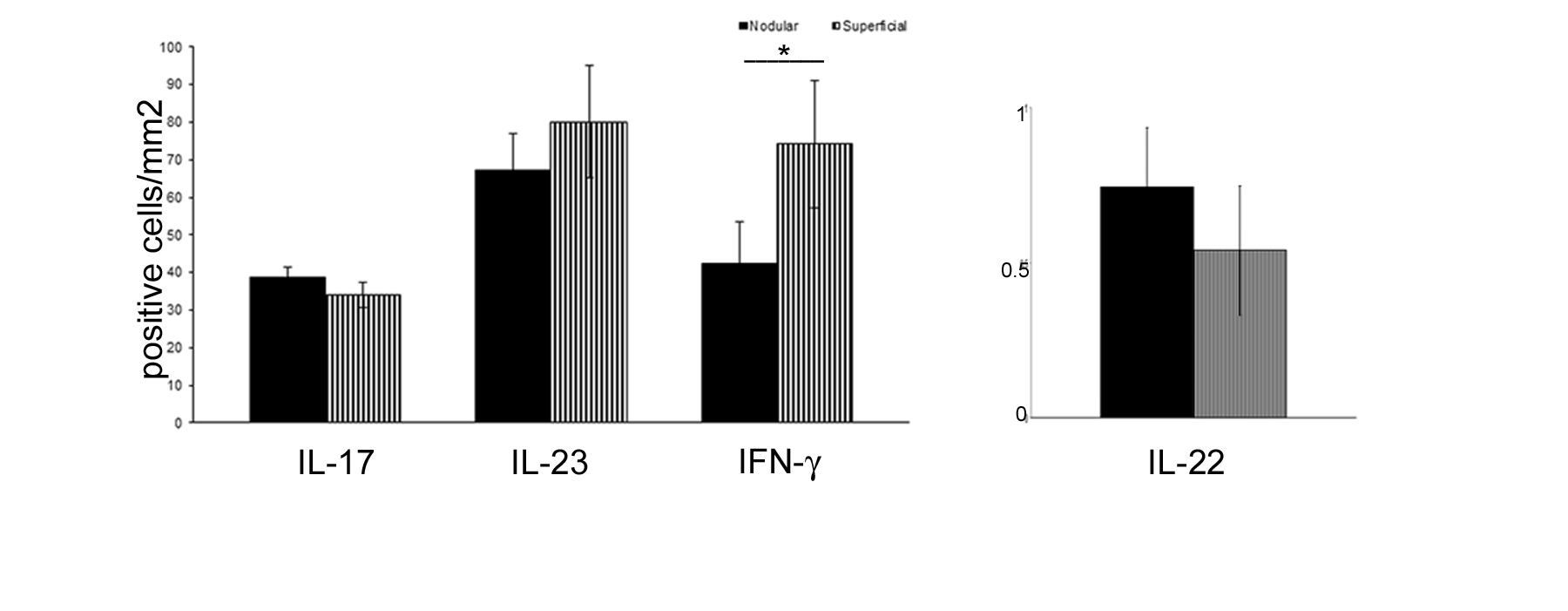

Supplement: S1 Fig — IFN-γ expression was significantly higher in sBCC as compared to nBCC (*p<0.05). A nonsignificant increase was also observed for IL-23. A trend for a higher expression of IL-17 and IL-22 was observed in nBCC compared to sBCC. (TIF) [file pone.0183415.s001.tif]

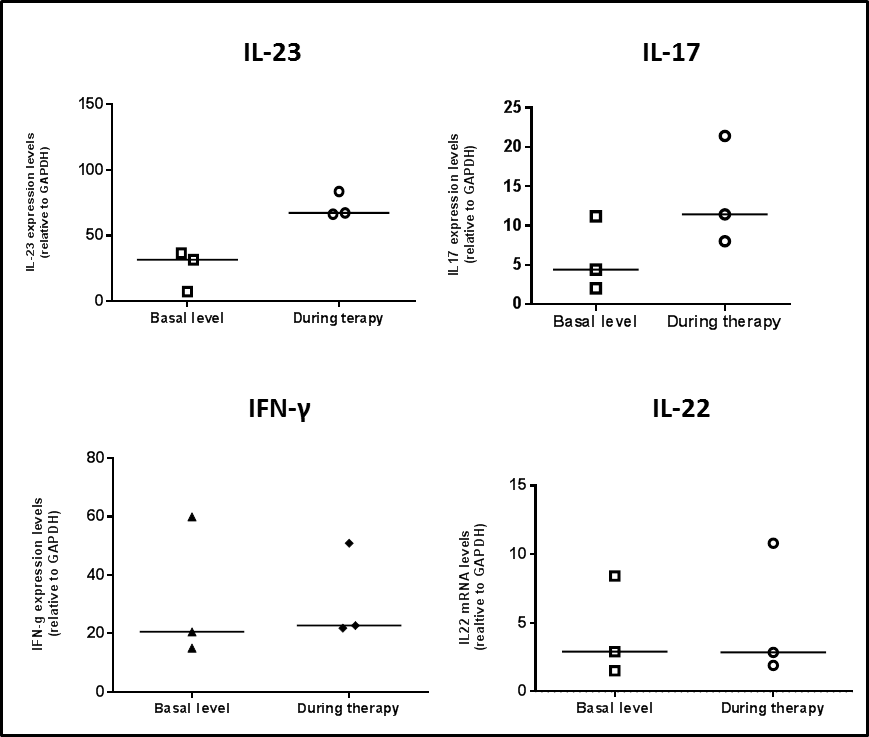

Supplement: S2 Fig — mRNA levels for IL-17 and IL-23 were higher during the inflammatory phase than at baseline while no difference was observed for IL-22 and IFN-γ. (TIF) [file pone.0183415.s002.tif]

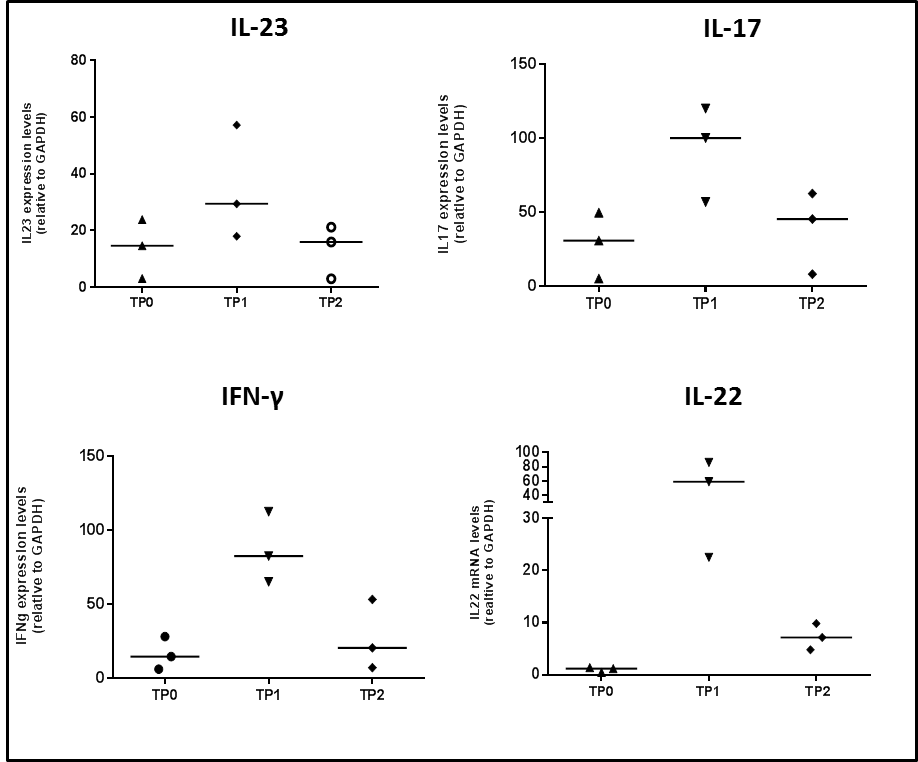

Supplement: S3 Fig — mRNA levels of all tested cytokines were increased at the early timepoint of MAL-PDT as compared to baseline followed by a decrease at the late timepoint. TP0, baseline; TP1, early time point; TP2, late time point. (TIF) [file pone.0183415.s003.tif]
